# Angular dispersion suppression in deeply subwavelength phonon polariton bound states in the continuum metasurfaces

**DOI:** 10.1038/s41566-025-01670-9

**Published:** 2025-05-16

**Authors:** Lin Nan, Andrea Mancini, Thomas Weber, Geok Leng Seah, Emiliano Cortés, Andreas Tittl, Stefan A. Maier

**Affiliations:** 1 Chair in Hybrid Nanosystems, Nanoinstitute Munich, Faculty of Physics, Ludwig-Maxilimians-Universität München, München, Germany; 2 School of Materials Science Engineering, Nanyang Technological University, Singapore, Singapore; 3 School of Physics and Astronomy, Monash University, Clayton, Victoria Australia; 4 Department of Physics, Imperial College London, London, UK

**Keywords:** Nanophotonics and plasmonics, Sub-wavelength optics, Polaritons

## Abstract

Quasi-bound states in the continuum (qBICs) achieved through symmetry breaking in photonic metasurfaces are a powerful approach for engineering resonances with high quality factors and tunability. However, miniaturization of these devices is limited as the in-plane unit-cell size typically scales linearly with the resonant wavelength. By contrast, polariton resonators can be deeply subwavelength, offering a promising solution for achieving compact devices. Here we demonstrate that low-loss mid-infrared surface phonon polaritons enable metasurfaces supporting qBICs with unit-cell volumes up to 10^5^ times smaller than the free-space volume $\lambda_{0}^3$. Using 100-nm-thick free-standing silicon carbide membranes, we achieve highly confined qBIC states with exceptional robustness against incident-angle variations, a feature unique among qBIC systems. This absence of angular dispersion enables mid-infrared vibrational sensing of thin, weakly absorbing molecular layers using a reflective objective, a method that typically degrades resonance quality in standard qBIC metasurfaces. We introduce surface-phonon-polariton-based qBICs as a platform for ultraconfined nanophotonic systems, advancing the miniaturization of mid-infrared sensors and devices for thermal radiation engineering.

## Abstract (web summary)

Phonon polariton quasi-bound states in the continuum realized in a dielectric metasurface patterned with a subwavelength lattice of elliptical holes in a commercially available free-standing, large-area 100-nm-thick silicon carbide membrane is demonstrated, attractive for applications in mid-infrared optics, such as molecular sensing and thermal radiation engineering.

## Main

Borrowed from a concept developed in quantum mechanics^1^, photonic bound states in the continuum (BICs) are localized states with formally infinite lifetimes at energies within the radiation continuum^2,3^. Although true BICs are dark modes, coupling to far-field radiation can be achieved by geometrical detuning of the structure from the BIC state, opening a radiative channel leading to the emergence of so-called quasi-bound states in the continuum (qBICs). Of particular interest are ‘symmetry-protected’ qBICs within metasurfaces, where coupling to the continuum results from the deliberate symmetry breaking of the unit-cell structure. These qBICs boast remarkable attributes, including high and tunable quality (*Q*) factors controlled by the asymmetry parameter^4,5^, readily adjustable resonance frequency through geometric size scaling^6^ and robustness against fabrication errors^7,8^. These compelling attributes have driven diverse applications of symmetry-protected qBICs, including but not limited to mid-infrared vibrational spectroscopy^6,9,10^, refractive-index sensing^11,12^, enhanced light–matter interactions^13–15^, photocatalysis^16^, lasing^17–19^ and thermal radiation engineering^20,21^

The *Q* factor of qBICs is governed by both radiation leakage (depending on the degree of asymmetry) and the inherent non-radiative decay due to material absorption. To enhance the *Q* factor, the most straightforward strategy is to utilize low-loss dielectrics with negligible intrinsic dissipation^6,15,22^. Recent endeavours have introduced plasmonic qBICs to amplify the surface field enhancement, although with reduced *Q* factors owing to increased losses from electron–electron scattering in noble metals^10,23^. Polar dielectrics supporting surface phonon polaritons (SPhPs) can effectively combine the benefits of both material platforms: high field enhancement due to the excitation of surface polaritons in the Reststrahlen (RS) band, and high *Q* factors due to the slow phonon–phonon scattering mechanism determining their decay^24–29^. Furthermore, qBICs in both dielectric and plasmonic metasurfaces typically feature lateral unit-cell sizes on the order of the resonance wavelength, hindering their miniaturization^30–32^. At the same time, the large size of the unit cell results in a strong spectral shift of the qBIC resonances when tilting the illumination angle as a consequence of the varying electromagnetic phase in adjacent unit cells^6,31,33^. Angular dispersion becomes particularly problematic when using broadband reflective Cassegrain objectives. The off-axis illumination of these objectives averages the metasurface response over a range of angles, reducing the *Q* factor. Consequently, qBIC metasurfaces for vibrational sensing have traditionally required specialized refractive objectives that are not standard in commercial Fourier transform infrared (FTIR) spectrometers and have reduced bandwidth due to material absorption^6,31,33^. Beyond enabling the use of Cassegrain objectives, angle-independent metasurfaces are required in advanced applications in which incident-light angles may vary. For example, in radar stealth technologies, maintaining a stable response across all angles is essential for optimizing absorption and minimizing reflection^34^. Similarly, such metasurfaces are vital for solar energy harvesting, water desalination and artificial colour generation, ensuring consistent performance irrespective of illumination or observation angle^35,36^. In on-chip sensing, angular robustness eliminates the need for additional optical elements, streamlining device integration^37,38^. These examples underscore the increasing demand for angle-robust metasurfaces across diverse wavelength ranges. For qBIC metasurfaces in particular, suppressing angular dispersion remains an active area of research, with recent advancements driven by the precise design and optimization of unit-cell geometries^39–41^.

Although deeply subdiffractional unit-cell volumes are theoretically possible in plasmonic qBICs, their practical implementation is hindered by considerable losses in the high-confinement region where Re(*ε*) ≲ 0. Only in this regime, a substantial portion of the electromagnetic field penetrates the material, and the polariton mode exhibits a strong ‘material-excitation’ character^42^, enabling electromagnetic-field confinement below the diffraction limit^43^. Owing to the intrinsic longer SPhP lifetimes, ‘phononic’ qBICs can efficiently operate in the subwavelength regime at frequencies where Re(*ε*) ≲ 0 (ref. ^44^). Although polaritonic qBICs have been recently shown in hexagonal boron nitride^45^, crucial conceptual aspects such as angular dispersion suppression, resonance and *Q*-factor tuning, and enhanced light–matter coupling have remained elusive, hindered by the limited scalability of metasurface fabrication on exfoliated flakes.

In this work, we demonstrate deeply subwavelength SPhP-driven symmetry-protected qBIC metasurfaces with a minimum unit-cell volume that is 10^5^ times smaller than the free-space volume ${V}_{0}={\lambda }_{0}^{3}$ at the resonant frequency. We fabricate the structures on commercially available large-area (up to few millimetres) free-standing complementary-metal–oxide-semiconductor-compatible silicon carbide (SiC) membranes^46,47^, where the *Q* factor is maximized owing to the symmetric environment provided by the absence of a solid substrate^48^. We confirm the qBIC character by amplitude- and phase-resolved maps obtained through transmission scattering-type scanning near-field optical microscopy (sSNOM). We demonstrate how the subwavelength nature of phononic qBICs eliminates the angular dispersion and subsequently leverage it to perform surface-enhanced vibrational sensing of a thin layer of spin-coated polymer with a standard Cassegrain objective. Polyethylene glycol (PEG) is chosen as the target molecule as it is an industrially relevant polymer that features a fingerprint vibrational mode lying within the SiC RS band. The combination of high *Q* factor and high field enhancement of phononic qBIC metasurfaces enables the realization of a vibrational strong coupling even with a weakly absorbing molecule such as PEG. Our work introduces phononic qBICs as a highly confined nanophotonic platform and paves the way for its implementation in mid-infrared (IR) applications such as molecular sensing^49–51^ and thermal radiation engineering^52–54^.

## Results

**Fig. 1:**
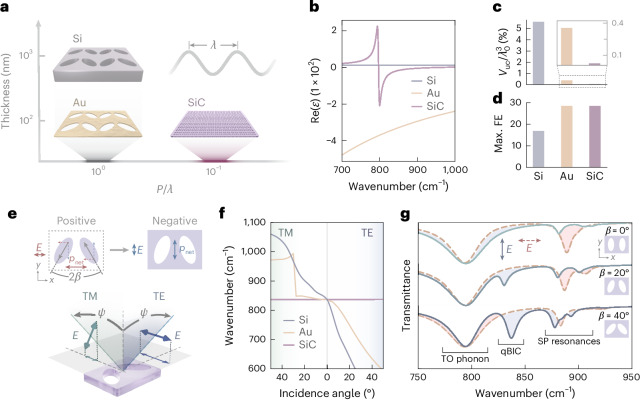
Highly confined SPhP-driven qBICs. **a**, Sketch of the unit cells for inverse metasurfaces made of Si (dielectric), Au (metal) and SiC (polar dielectric), showing a qBIC resonance at *λ* = 11 µm. The horizontal axis is the characteristic edge length of the unit cell compared with the wavelength, and the vertical axis indicates the structure thickness. **b**, Real part of the permittivity for Si, Au and SiC. Comparison of the unit-cell volume *V*_uc_ (**c**) and the maximum field enhancement (FE) (**d**) for the three metasurfaces at the qBIC resonance (the inset in **c** shows the zoomed-in view of only Au and SiC). **e**, For a negative metasurface, the polarization required to excite the qBIC is along the short unit-cell axis (*y* direction), rotated by 90° compared with the positive structure, as dictated by the Babinet principle. **f**, Simulated qBIC wavenumber for Si, Au and SiC for the negative case from **e**. The SiC qBIC displays no angular dispersion for both TM and TE polarizations, whereas the Si and Au counterparts heavily shift with tilted illumination. **g**, Simulated spectra for a SiC negative metasurface at normal incidence with different ellipses tilting at angles *β* under excitation with polarization along the *y* (solid curves) and *x* (dashed curves) directions. SP indicates resonances present in single holes (that is, single particles).

To highlight the advantages brought by our BIC-driven SiC phononic metasurface, we compare it with analogous dielectric and metal structures, namely, silicon (Si) and gold (Au) supporting a qBIC resonance at the same wavelength (~11.2 µm; Supplementary Discussion 1). We consider inverse (or ‘negative’) metasurfaces composed of elliptical perforations milled into a film (either Si, Au or SiC) in which the radiative channel to enable the far-field coupling of the qBIC is activated by the tilting of the ellipses with respect to each other^6^. The most striking difference between the three materials is in the unit-cell volume (Fig. 1a), where the SiC structures are remarkably smaller. The real parts of the permittivity for the dielectric, metallic and phononic cases are shown in Fig. 1b. For the dielectric metasurface (with Re(*ε*_Si_) ≈ 10), the lateral size of the unit cell, although subwavelength, is typically comparable with the resonance wavelength, and its minimum thickness is on the order of half a micrometre. The metallic, that is, Au, structure allows for a thickness reduction by one order of magnitude. However, the lateral size remains similar to that of the dielectric metasurface because at mid-IR frequencies, noble metals behave similar to perfect electric conductors^55^ (Re(*ε*_Au_) ≪ 0). By contrast, our 100-nm-thick SiC metasurface supports qBIC resonances even for deeply subwavelength unit cells due to the negative, near-zero permittivity value (Re(*ε*_SiC_) ≲ 0), allowing extreme miniaturization of the device. The quantitative comparison of the unit-cell volume obtained from full three-dimensional electromagnetic simulations (Fig. 1c and Methods) shows that the Au unit cell is an order of magnitude smaller than that of Si, whereas the SiC unit cell is a further order of magnitude smaller. Moreover, the maximum field enhancement at resonance is approximately twice as large in both plasmonic and phononic metasurfaces compared with that reached in the dielectric structure (Fig. 1d).

In conventional ‘positive’ qBIC metasurfaces (made of antennae placed on a substrate), the net dipole moment *p*_net_ induced by the antennae tilting is along the *x* direction of the unit cell due to the rotation of the opposite dipoles in the resonators (Fig. 1e). Consequently, to excite the qBIC, the polarization of the excitation beam has to be along the *x* direction. According to Babinet’s principle, in a negative metasurface (that is, holes in an extended film), the polarization has to be flipped by 90° (ref. ^31^) (Fig. 1e). A key feature of phononic qBICs is the robustness of the resonance frequency against tilt in the illumination direction (Fig. 1e). Since mid-IR devices are generally probed using reflective Cassegrain objectives characterized by off-normal illumination, the performance of qBIC metasurfaces can be compromised by the inherent angular spread^31^. The deep-subwavelength nature of phononic qBICs means that even under tilted illumination, the local phase of the electric field is indistinguishable between adjacent unit cells, resulting in unique angular stability of the resonance^33^. The qBIC mode can be excited under both transverse-magnetic (TM) and transverse-electric (TE) polarizations when the unit cell of the metasurface is properly oriented. Although the phononic SiC metasurface exhibits dispersionless behaviour for both polarizations, both dielectric and metallic structures suffer from considerable angular dispersion (Fig. 1e,f). For the case of TM dispersion in the Au structure, the noticeable jump in dispersion can be attributed to coupling with a grating mode that crosses the qBIC resonance (Supplementary Discussion 1).

For the practical realization of phononic qBICs, we chose a commercially ready complementary-metal–oxide-semiconductor-compatible free-standing SiC membrane. The simulated transmission spectra for a SiC metasurface at various ellipses tilting angles *β* are shown in Fig. 1e. Each spectrum is computed for two polarizations, aligned with the two orthogonal edges of the unit cell. Although a thick SiC layer (several micrometres) is highly reflective in the RS band^25,28^, our 100-nm-thick membranes are below the skin depth and, thus, highly transparent^46^, except for a dip around 800 cm^*−*1^ due to the transverse-optical (TO) phonon absorption. In the band from 875 cm^*−*1^ to 925 cm^*−*1^, we observe a series of dips for both polarizations and for all *β*, which we associate with modes existing in the single elliptical perforations. At 830 cm^*−*1^, we observe a resonance that is associated with a qBIC as it disappears for *β* = 0° and exists only for one of the polarizations^6,7,22^.

**Fig. 2:**
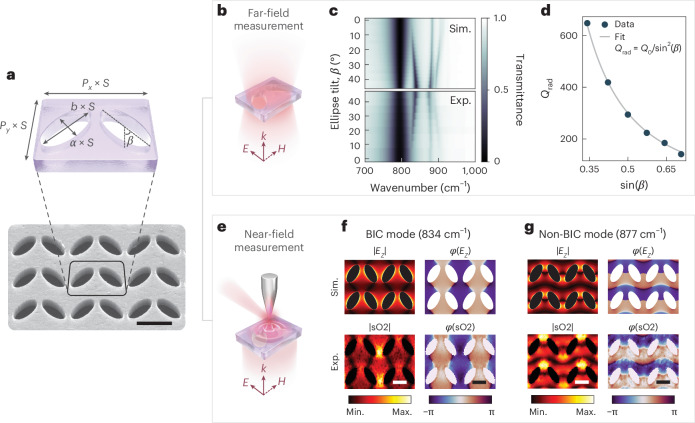
Far- and near-field characterizations of SPhP-driven qBICs. **a**, Illustration of the metasurface unit cell with relevant geometrical parameters (top) and tilted scanning electron microscopy image of a portion of the fabricated array (bottom). Scale bar, 500 nm. **b**, Sketch of the polarization direction in the transmission FTIR measurements. **c**, Simulated (top) and experimental (bottom) far-field transmission spectra for a metasurface with *S* = 1 and varying tilting angle *β*. **d**, Radiative *Q* factor extracted from the TCMT fit of the experimental data in **c**. **e**, Sketch of the sSNOM setup for the near-field mapping. **f**, Simulated (top) amplitude (left) and phase (right) of the out-of plane component of the surface electric field at the qBIC resonance frequency. The near-field amplitude and phase measured with sSNOM (second demodulation order, indicated as sO2) are shown in the bottom. **g** Same as in **f**, but at a frequency of a higher-order mode that does not show a qBIC character. Scale bar, 500 nm.

We confirm the realization of qBIC states in SiC metasurfaces through a combination of far- and near-field experiments. The fabrication process begins with a standard electron-beam lithography step for the creation of a chromium hard mask, followed by a reactive ion etching treatment to mill holes into the membranes (Methods). A sketch of a single unit cell with relevant geometrical parameters is shown in Fig. 2a together with a scanning electron microscopy image of a portion of one of the fabricated arrays. The long and short axes of the ellipses are set to be *a* = 1,190 nm and *b* = 560 nm, whereas the edges of the unit cell are *P*_*x*_ = 2,100 nm and *P*_*y*_ = 1,610 nm. These dimensions are defined as the scaling factor *S* = 1. Although adjusting the thickness of the material offers a means to tune the position of the qBIC resonance frequency (Supplementary Discussion 2), altering the scaling factor is a more convenient and efficient method for tuning the resonance of qBIC metasurfaces^6,22^. The theoretical spectra (Fig. 1e) are validated through transmittance measurement with FTIR microscopy (Methods). To demonstrate the existence of qBIC resonance, we maintain all the geometrical parameters of the unit cell as constant but solely vary the tilting angle *β* between the elliptical holes. Following Babinet’s principle, we polarize the electric field of the incident beam along the short axis of the unit cell^31^ (Fig. 2b). When changing the ellipse tilting angle *β*, we observe a series of dips in both simulated (top) and experimental (bottom) transmission spectra (Fig. 2c). The lowest-energy dip is associated with the TO phonon absorption from the bare SiC membrane. At higher energy, the qBIC resonance can be identified as it becomes optically active only for *β* *>* 0°. As expected, we observe an increase in the modulation depth and linewidth of the resonance with increasing *β* due to the strengthening of the radiative dissipation channel introduced by the structure asymmetry. The achieved total *Q* factors (Supplementary Discussion 4) align well with the reported values of high-*Q* phonon polariton resonances in structured SiC metasurfaces^25^. Higher-energy dips are associated with higher-order resonances of the elliptical meta-atoms as they are present even at *β* = 0°. The close match between the experimental and simulated spectra confirms the accuracy of our theoretical model and interpretation. Interestingly, we observe a narrowing of the TO phonon bandwidth with increasing *β* and qBIC modulation depth (Supplementary Discussion 3), indicating the presence of coupling between the two.

To further confirm the qBIC characteristic of the resonance, we fit the experimental spectra with a temporal coupled-mode theory (TCMT) model (Supplementary Discussion 4). From this fit, we can extract the radiative part of the resonance *Q* factor *Q*_rad_ (Fig. 2d). As a general feature of symmetry-protected BIC-driven metasurfaces, *Q*_rad_ is proportional to the inverse square of the asymmetry parameter, which—for a metasurface made of elliptical antennae—can be defined as sin^2^(*β*) (ref. ^4^). The *Q*_rad_ values extracted from the TCMT model fit well with the 1/sin^2^(*β*) function, confirming the qBIC character of the resonance.

To study the field-enhancement distribution and ultimately confirm the qBIC nature of the observed resonance, we carry out nanoscale mapping using a scattering sSNOM system (Fig. 2e). We perform the measurements in the transmission mode, which is ideal for the investigation of resonators due to reduced tip–sample coupling and normal-incidence illumination^56,57^. Briefly, a mid-IR beam from a tunable mid-IR laser source is focused from below by a parabolic mirror on the tip of an atomic force microscope. The tip scatters the incident light, which is collected by an off-axis upper parabolic mirror. The signal is demodulated at higher harmonics (*n* ≥ 2) of the tip oscillation frequency for far-field background suppression and is analysed using a pseudo-heterodyne interferometer for extracting the amplitude and phase responses (Methods).

For the near-field mapping, we use a metasurface with *S* = 1 and *β* = 30°; its far-field response is shown in Fig. 2c. Results for frequencies at which the structure supports a qBIC (*ω* = 834 cm^*−*1^) and a single elliptical hole resonance (*ω* = 877 cm^*−*1^) are reported in Fig. 2f,g, respectively. For each frequency, the top row shows the simulated amplitude (left) and phase (right) of the out-of plane component of the field *E*_*z*_ at the SiC surface. The corresponding near-field data (demodulation order, *n* = 2) are shown in the bottom row, demonstrating good agreement between the simulated and experimental maps. A small discrepancy can be seen in the experimental amplitude maps, where high-field hot spots appear at the antenna gap that have no counterpart in the simulation. We attribute this effect to the combination of non-perfect normal illumination and residual tip–sample coupling, which can impact the measured amplitude, but have negligible effect on the phase profile. We use the topography to mask the optical data in the region of the perforations, where the mechanical tip–sample interaction is unreliable due to the absence of a substrate. Interestingly, we can clearly distinguish the qBIC resonance (Fig. 2f) as the phase jumps are in the *x* direction and perpendicular to the *y* polarization of the exciting beam. This effect is a direct consequence of the qBIC nature: in the symmetric structure, the true BIC corresponds to opposing horizontal dipoles (along the short ellipses axis) that mutually cancel in the far field. When breaking the symmetry, the dipoles acquire vertical components that do not cancel out, giving rise to a net dipole moment in the *y* direction, which allows mode coupling to the far-field illumination. The out-of-plane field components align with those of the in-plane direction, pointing outwards from the structure at one side of the ellipses and inwards at the other one. This creates the horizontal phase jumps observed in *E*_*z*_ (Supplementary Discussion 5 provides a more extensive discussion using simulated data). For the non-qBIC mode shown in Fig. 2g, the phase jumps are, as expected for a dipolar-type mode, along the polarization direction (in the *y* direction). The combination of far- and near-field measurements (Fig. 2) allows us to characterize and unequivocally confirm the qBIC nature of the deeply subwavelength resonances we engineered in our SiC phononic metasurfaces.

**Fig. 3:**
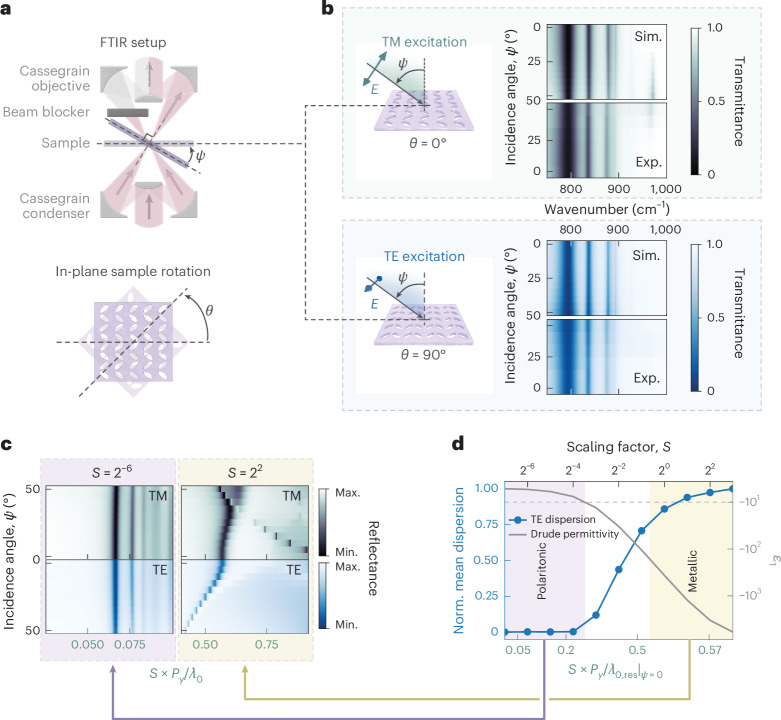
Angular robustness of deeply subwavelength qBICs. **a**, Experimental setup for transmission measurement with various incidence angles *ψ*. **b**, Simulated and experimental spectra for a metasurface with *S* = 1 and *β* = 40° under effectively tilted illumination at multiple angles from normal incidence to *ψ* = 50°. Plots are shown for both TM (top, *θ* = 0°) and TE (bottom, *θ* = 90°) polarizations. **c**, Simulated angular response for a metasurface made out of an idealized Drude material. For a small scaling factor (*S* = 2^*−*6^), there is no angular dispersion, whereas angular robustness is lost for a large unit-cell size (*S* = 2^2^). Instead of plotting against the frequency, here we use the normalized quantity *S* × *P*_*y*_/*λ*_0_ to highlight the ratio of the unit-cell size to the free-space wavelength. **d**, Average derivative of the angular dispersion as a function of the unit-cell size for an idealized Drude model. Although large unit cells (in the metallic regime) have pronounced angular dispersion, deeply subwavelength unit cells (in the polaritonic regime) show excellent angular robustness. In the same plot, we also report the value of the real part of the dielectric function at the qBIC resonance frequency for each *S*.

In conventional qBIC systems (both plasmonic and dielectric), the resonance frequency is heavily dependent on the illumination angle^31,33^. For this reason, refractive objectives with low numerical apertures are generally required to obtain good modulation depths in qBIC systems. A standout feature of our phononic qBIC is its dispersionless nature, which can already be indirectly inferred from the pronounced resonance dips we observe in our far-field spectra (Fig. 2c) obtained with a Cassegrain objective. As illustrated in Fig. 3a, the illumination from a Cassegrain objective averages the metasurface response over a spread of off-axis angles, leading to a decrease in the resonance quality for any photonic structure with angular dispersion^31^. To confirm this angular robustness, we mount our samples on a tilting stage, allowing us to measure the transmission spectra for various incidence angles with respect to the sample plane (Fig. 3a). For this, we partially block the upper objective and probe the responses of the metasurfaces with a spread of angles averaged over a single value when tilting the sample. By rotating the polarization by 90°, we can measure both TM and TE responses. To ensure the excitation of the qBIC (electric field along the short unit-cell axis), we rotate the metasurfaces in plane by an angle *θ* so that for *θ* = 0°, the qBIC is activated for TM polarization, whereas for *θ* = 90°, it is activated for TE polarization (Fig. 3a). Strikingly, simulations and measurements for a metasurface with *S* = 1 and *β* = 40° demonstrate the clear independence of all the modes to the illumination angle *ψ* (Fig. 3b). The lack of angular dispersion is observed for both TM (for *θ* = 0°, top panels) and TE (for *θ* = 90°, bottom panels). In particular, the consistent qBIC resonance positions and their unvarying modulation depths underscore the superior angular robustness of our system. The only appreciable variation in the spectra can be seen around 970 cm^*−*1^ due to the excitation of the so-called Brewster mode at the epsilon-near-zero point^58^. This interpretation is confirmed by the appearance of this transmission dip only for TM-polarized excitation (Supplementary Discussion 6). We attribute the angular robustness of our design to the unique deeply subwavelength character of the phononic qBIC, which implies that the neighbouring unit cells are illuminated with an almost-identical phase even for highly tilted illumination.

To support our interpretation, larger unit-cell sizes in SiC metasurfaces could be investigated as they should eventually lead to the appearance of angular dispersion. However, increasing the scaling factor redshifts the qBIC resonance closer to the TO phonon that leads to coupling between the two modes, which makes a clear observation of frequency shifts complicated. Therefore, we numerically investigate the angular dispersion in a simplified material described by a Drude model with no interband transitions (Supplementary Discussion 8). We maintain the same metasurface geometry and simulate the optical response for TM and TE polarizations (Fig. 3c). In the left panel, we report the calculated spectra for a small unit-cell size corresponding to *S* = 2^−^^6^, where there is no angular dispersion as that for the SiC qBIC case. For a large unit cell (*S* = 4, right panel), we instead recover a response similar to the one of a Au metasurface at mid-IR frequencies (Fig. 1e) for which dispersion appears for both TM and TE polarizations. Given the widely different unit-cell sizes, the resonances for the two different *S* values lie at very different frequencies. To highlight this effect, instead of plotting the spectra against the frequency in wavenumbers 1/*λ*_0_, we use the short side of the unit cell *S* × *P*_*y*_ normalized by the free-space wavelength *λ*_0_ as the *x* axis. For metallic and dielectric qBICs, it is often assumed that the resonance frequency shifts linearly with *S* (ref. ^6^). Therefore, the ratio *S* × *P*_*y*_/*λ*_0_ at which the qBIC appears should remain constant. However, when dealing with a material described by either a Drude or Lorentz model, the linear relationship between the qBIC frequency and the scaling factor breaks down when approaching frequencies close to the transition from a negative to a positive dielectric function. At these frequencies, just below the plasma frequency for metals, the optical response is expected to feature strong polaritonic effects, showing a saturation of the frequency shift, deeply subwavelength unit cells and loss of angular dispersion (Supplementary Fig. 15a). To fully grasp the transition from ‘metallic’ to ‘polaritonic’ behaviour, we performed various simulations for different *S* values. For each *S*, we calculate the average absolute derivative of the qBIC frequency against the incidence angle, which gives us a figure of merit quantifying the angular dispersion (Fig. 3d). We focused on the TE dispersion because at high *S*, the TM mode couples with the grating mode; however, the behaviour is essentially the same for both polarizations (Supplementary Fig. 14). As the unit-cell size shrinks, the angular dispersion strongly drops. At the same time, we plot, on the right axis, the value of the dielectric function at the qBIC resonance, showing how the angular dispersion is lost for Re(*ε*) *>* −10, corresponding to the polaritonic regime. The normalized unit-cell size *S* × *P*_*y*_/*λ*_0_ allows us to draw a comparison with the SiC and Au qBICs at mid-IR frequencies. For all the SiC metasurfaces investigated, *S* × *P*_*y*_/*λ*_0_ *<* 0.2, confirming that they are in the polaritonic regime in which angular dispersion is not anticipated. On the other hand, Au metasurfaces with a qBIC resonance within the SiC RS band have *S* ≈ 4 with *S* × *P*_*y*_/*λ*_0_ ≈ 0.5, placing them in the metallic regime. To confirm that the behaviour we observe here is not associated with the particular unit-cell geometry, we simulated a positive phononic qBIC metasurface that also exhibits angular robustness. At the same time, the Au metasurface is strongly dispersive in both positive and negative configurations (Supplementary Discussion 7). These observations exclude the attribution of the angular independence to the particular metasurface geometry and point to the generality of the phenomena for subwavelength unit cells. However, angular dispersion suppression through subwavelength unit-cell scaling cannot be easily achieved in conventional plasmonic or dielectric systems. For metals, losses close to the plasma frequency due to interband transitions prevent the fabrication of high-quality subwavelength resonators. Doped semiconductors could be an alternative at mid-IR frequencies, but their high damping rates lower the achievable field enhancement and *Q* factor^43^. In principle, dielectric metasurfaces made of high-index transparent materials could be resonant at subwavelength unit-cell scales *λ*_0_/*n*; however, there are fundamental limitations that prevent the increase in refractive index without substantial losses^59^, and such high-index materials are not currently available. This renders our SiC metasurfaces a standout platform for angle-robust qBICs.

**Fig. 4:**
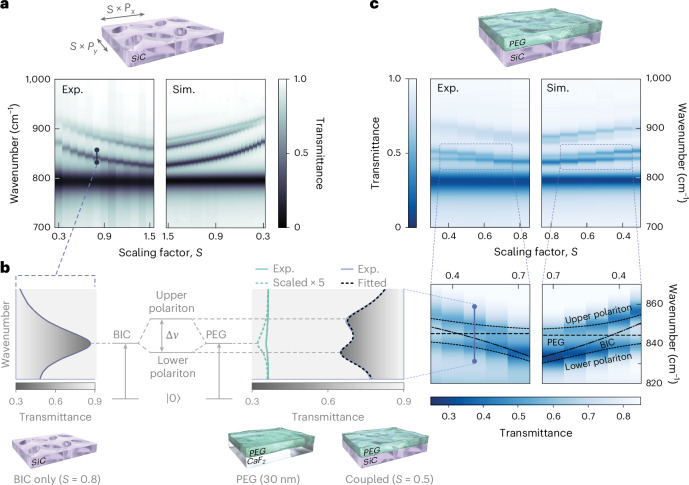
Vibrational strong coupling of SiC qBICs with PEG. **a**, Experimental and simulated transmission spectra of bare SiC metasurfaces with scaling factors ranging from 0.4 to 1.5. **b**, Schematic of vibrational strong coupling. The BIC resonance is spectrally overlapped with the absorption band of PEG, resulting in a splitting of the BIC mode into upper and lower polaritonic states. **c**, Experimentally resolved and simulation-resolved anticrossing of SiC metasurfaces covered with 30-nm-thick PEG. The dotted lines denote the polariton dispersion calculated using the extracted parameters from TCMT fitting and a Rabi splitting of 11.5 cm^*−*1^.

The unique angular stability of qBIC metasurfaces can be leveraged to perform mid-IR vibrational surfaced-enhanced infrared absorption without the need for specific refractive objectives. Moreover, the combination of high *Q* factor and high field enhancement is expected to yield strong light–matter interactions. We select PEG as a prototype molecule that features a characteristic C–H bending mode at *ω* = 842 cm^*−*1^, matching SiC’s RS band. PEG is a technologically relevant water-soluble polyether compound widely used in pharmaceutical, cosmetics and manufacturing industries^60^. To match the qBIC and PEG resonance frequencies, we study the tunability of our metasurfaces via *S* tuning. As the phononic qBIC resonance can only exist inside the material RS band, we observe (Fig. 4a) a highly nonlinear frequency shift in the experimental (left) and simulated (right) transmission spectra of a metasurface with *β* = 40°. As discussed in the previous section, a nonlinear shift in the qBIC frequency is expected for any system operating in the polaritonic regime. The smallest metasurface fabricated for this study features *S* = 0.25, corresponding to *S* × *P*_*y*_/*λ*_0_ = 0.033, deeply in the polaritonic regime and with a unit-cell volume of 1.25 × 10^−5^—smaller than the free-space volume. Importantly, the qBIC tunability extends both above and below the PEG vibrational mode, allowing a comprehensive investigation of the light–matter coupling strength.

To enable effective near-field interaction, we spin coat our SiC metasurfaces with a layer of PEG with an estimated thickness of 30 nm (Supplementary Discussion 10). The effect of the PEG layer on the qBIC resonance is shown in Fig. 4b. When the frequencies of both modes overlap, a splitting of the qBIC dip can be observed in the transmission spectra, corresponding to the creation of two polariton branches of different energies. For numerical modeling and fitting of the coupled states, the dielectric function of PEG was calculated from Kramers–Kronig-consistent fits to the transmission measurements of films of different thicknesses on a CaF_2_ substrate. The extracted linewidth of the PEG absorption band was *γ*_PEG_ = 3.59 cm^*−*1^, which we fix for the TCMT fits used to extract the coupling strength. In Fig. 4c, we report the experimental (left) and simulated (right) transmission spectra for the coupled system when varying the metasurface scaling factor. Two changes in the optical response of our SiC metasurface can be observed: (1) a spectral redshift of the qBIC resonances due to the change in the local refractive index of the environment, resulting in a reduction in the scaling factor needed to achieve spectral overlap between qBIC and PEG; (2) a band splitting at the absorption peak of PEG around 842 cm^*−*1^ into the upper and lower polaritonic branches (Fig. 4b). Notably, the absorption fingerprint of the molecule on the metasurfaces is strongly enhanced compared with the bare CaF_2_ substrates, which can be attributed to the pronounced near-field enhancement of the qBIC mode. Detuning the qBIC mode from the PEG absorption by varying the scaling factor *S* reveals the typical anticrossing pattern characteristic of strongly coupled resonances. In the numerical simulations, we fill the elliptical holes of the membrane completely and add a 30-nm-thick layer on the top side of the membrane, resulting in excellent agreement with our experimental results. To quantify the strength of the vibrational coupling, we fit transmittance spectra with our TCMT model (Supplementary Discussion 4.3 provides more information on the fitting procedure), which allows for the extraction of the dispersion and linewidth of the qBIC from the coupled spectra, which would be unattainable from mere polariton dispersion fits.

As shown in Supplementary Fig. 8, the extracted qBIC dispersion from simulations follows a similar quadratic and continuous dependence on the scaling factor that we observe from the experimental measurements of bare metasurfaces. Because the thickness of the PEG varies slightly depending on the scaling factor in the experiment, we instead approximate the qBIC dispersion with a linear relationship. Furthermore, we simplify the system by assuming a constant qBIC linewidth over the sampled scaling-factor range, which is consistent with commonly utilized fits to extract the Rabi splitting of anticrossing spectra. The damping rate of the qBIC is extracted to be *γ*_BIC_ = 7.57 cm^*−*1^ and the near-field coupling strength at the point of spectral overlap between qBIC and PEG to be *g* = 6.08 cm^*−*1^ via interpolation at a scaling factor of *S* = 0.435. For the system to be in the strong-coupling regime, the exchange of energy between qBIC and PEG needs to be faster than the constituting resonances can dissipate energy into their individual loss channels^13,61^. In other words, the frequency separation of the polariton peaks (the vacuum Rabi splitting 2*g*) must be larger than the sum of the linewidths of BIC and PEG. This is commonly referred as the strong-coupling criterion *c*_1_ = 2*g*/(*γ*_BIC_ + *γ*_PEG_) *>* 1, which we evaluate for our system to be *c*_1_ = 1.09. Because of the mismatch of the BIC and PEG linewidths, the peak splitting is actually given by the Rabi splitting ${{\varOmega}}_{\rm{R}}=2\sqrt{{g}^{2}-\left({\gamma}_{\rm{BIC}}-{\gamma}_{\rm{PEG}}\right)^{2}/4}=11.5\,{\rm{cm}}^{-1}$, which is slightly lower than 2*g* and a stricter criterion is given by *c*_2_ = *Ω*_R_/(*γ*_BIC_ + *γ*_PEG_) *>* 1 with *c*_2_ = 1.03 (Supplementary Discussion 4.3). Both criteria are fulfilled and show that we reach the strong-coupling regime. Finally, we want to remark that we are able to reach the strong-coupling regime even with the relatively weak vibrational peak of PEG when compared with molecules routinely used for mid-IR vibrational strong coupling such as SiO_2_ and polymethyl methacrylate^62–65^ (Supplementary Discussion 12). These observations highlight the potential of phononic qBICs for the investigation and exploitation of mid-IR strong coupling at the nanoscale.

## Conclusions

In this work, we reported the pioneering realization of a phonon-polariton-mediated qBIC metasurface. The qBIC nature was unequivocally assessed through comprehensive far- and near-field characterizations, supported by rigorous numerical simulations. By utilizing a low-loss polaritonic material, we achieved extreme miniaturization by shrinking the unit-cell size down to ~1.25 × 10^−5^ *×* $\lambda_{0}^3$, where $\lambda_{0}^3$ is the volume occupied by the free-space wavelength. This remarkable miniaturization led to the observation of a striking incident-angle-invariant behaviour on our metasurfaces, a highly unconventional characteristic for a symmetry-protected qBIC metasurface^31,33^. Using numerical simulations, we confirmed how this behaviour is a general feature of deeply subwavelength polaritonic metasurfaces, independent of the specific material or resonator geometry. This result paves the way for the utilization of phononic qBIC metasurfaces, and subwavelength polaritonic systems in general, in applications requiring consistent performances over a wide range of incidence angles such as in stealth technologies^66^ and omnidirectional radiative cooling^67,68^. We also showcase how the lack of angular dispersion allows us to perform surface-enhanced infrared absorption experiments with a standard off-axis Cassegrain objective without any degradation of the qBIC resonance quality. The strong electromagnetic confinement facilitated the emergence of vibrational strong coupling between a weak resonator (for example, PEG) and our qBIC system. Although phonon polariton vibrational strong coupling has been shown with hBN antennae obtained from exfoliated flakes^50^, here we report its realization in a large-area complementary-metal–oxide-semiconductor-compatible platform. The use of SiC is, therefore, suited for direct applications in surface-enhanced infrared absorption^69,70^ and nonlinear mid-IR optics^71,72^, as well as for the control of chemical reaction pathways through strong-coupling engineering^73,74^ at the nanoscale.

## Methods

### Sample preparation

Metasurfaces were fabricated on free-standing 100-nm SiC membranes supported by a Si frame purchased from Silson. Fabrication follows the conventional top-down nanofabrication method. First, the electron-beam resist, SX AR-N 8200.06, was exposed with a 10-µm aperture under 30-kV acceleration voltage, followed by the deposition of chromium as a hard mask for reactive ion etching via electron-beam deposition. SF_6_-based reactive ion etching was performed to perforate the SiC membrane followed by a wet-etching process for the removal of the hard mask.

For the vibrational coupling measurements, PEG 2000 was purchased from Sigma-Aldrich and dissolved into methoxybenzene to achieve a 12.5 mg ml^–1^ solution. The PEG solution was spin coated onto the suspended SiC metasurfaces. The PEG thickness on the SiC metasurfaces was estimated by interpolating the thickness–absorption correlation curve acquired from ellipsometry and FTIR spectroscopy on bulk substrates (Supplementary Discussion 10.)

### FTIR spectroscopy

Transmission spectra of the metasurfaces were acquired using a VERTEX 80v FTIR spectrometer (Bruker) in conjunction with a HYPERION 3000 microscope (Bruker). Measurements were performed using a reflective microscope objective (Newport), also known as Cassegrain objective, featuring a numerical aperture of 0.4 and ×15 magnification covering a range of polar angles from 12° to 23.6°. A liquid-nitrogen-cooled mercury cadmium telluride detector recorded the spectra in the transmission mode. The sample tilting was achieved by mounting the sample on a custom-made sample holder that can be rotated along the central axis that is perpendicular to the effective beam path. Custom-made aperture was added onto the objective over half of the transmitted beam for the tilted measurements.

### Transmission sSNOM

Near-field spectra were obtained using a commercial sSNOM setup (Neaspec) equipped with a pseudo-heterodyne interferometer to obtain the amplitude- and phase-resolved images. The light source used in the experiments was an optical parametric oscillator laser (Stuttgart Instruments) powered by a pump laser at *λ* = 1,035 nm with 40-MHz repetition rate and ~500-fs pulses. The linearly polarized mid-IR output was obtained by difference-frequency generation in a nonlinear crystal between the signal and idler outputs of the optical parametric oscillator. The beam frequency bandwidth was reduced using a monochromator. The beam was loosely focused at normal incidence by a parabolic mirror positioned below the sample. The near-field signal was scattered by a metal-coated (Pt/Ir) atomic force microscope tip (Arrow-NCPt, Nanoworld) oscillating at a frequency of *Ω* ≈ 280 kHz, and collected by a second off-axis parabolic mirror positioned above the sample. The tapping amplitude was set to ~80 nm and the signal was demodulated at the second-harmonic 2*Ω* for background suppression. Before focusing, half of the light was redirected towards a pseudo-heterodyne interferometer used to retrieve both amplitude and phase of the signal. The light scattered by the tip was recombined with the interferometer reference arm by a second beamsplitter and directed towards a nitrogen-cooled mercury cadmium telluride IR detector.

### Numerical simulations

Electromagnetic simulations were performed with a commercial real-space, full-wave, finite-element Maxwell solver (CST Studio Suite 2021) in the frequency domain. We used tetrahedral meshing with automated mesh refinement, which was convergence tested to guarantee accurate results. The metasurface response was simulated using periodic Floquet boundary conditions and linearly polarized plane-wave excitation. The electric-field distributions were extracted at the top layer of the SiC film.

The dielectric function of SiC was modelled as follows:

1 $$\varepsilon \left(\omega \right)={\varepsilon }_{\infty }\left(\frac{1+{\omega }_{{{\rm{LO}}}}^{2}-{\omega }_{{{\rm{TO}}}}^{2}}{{\omega }_{{{\rm{TO}}}}^{2}-{\omega }^{2}-{\rm{i}}\gamma \omega }\right),$$

where *ω*_TO_ = 797 cm^*−*1^, *ω*_LO_ = 973 cm^*−*1^, *ε*_∞_ = 6.6 and *γ* = 6.6 cm^*−*1^. The PEG dielectric function was modelled with a single Lorentzian as

2 $${\varepsilon }_{{\rm{PEG}}}^{\left(\omega \right)}={\varepsilon }_{{\rm{PEG}},\infty }+\frac{{A}_{{\rm{PEG}}}}{{{\omega }_{2}}_{{\rm{PEG}},0-{\omega }^{2}-2{\rm{i}}{\gamma }_{{\rm{PEG}}}\omega }},$$

where *ε*_PEG,∞_ is set to 2.25, *ω*_PEG,0_ = 842.4 cm^*−*1^, *γ*_PEG_ = 3.59 cm^*−*1^ and the oscillator strength *A*_PEG_ = 8,026 cm^*−*2^. The factor of two in the denominator is due to the convention chosen to be comparable with our TCMT model, where *γ* refers to the half-width at half-maximum of a Lorentzian.

## Online content

Any methods, additional references, Nature Portfolio reporting summaries, source data, extended data, supplementary information, acknowledgements, peer review information; details of author contributions and competing interests; and statements of data and code availability are available at 10.1038/s41566-025-01670-9.

## Supplementary information

Supplementary Information Supplementary Figs. 1–18, Discussions 1–12 and Tables 1 and 2.

## Data Availability

The data supporting the findings of this study are available in the Article and its Supplementary Information, as well as via Zenodo at 10.5281/zenodo.14965599 (ref. ^75^). Additional data are available from the corresponding authors upon reasonable request.
