# Supplementary material for: Angular dispersion suppression in deeply subwavelength phonon polariton bound states in the continuum metasurfaces
Source: Nat Photonics. 2025 May 16;19(6):615–23. doi: 10.1038/s41566-025-01670-9 (PMC12141033; doi:10.1038/s41566-025-01670-9)
Supplement: Supplementary file 1 — Supplementary Figs. 1–18, Discussions 1–12 and Tables 1 and 2. [file 41566_2025_1670_MOESM1_ESM.pdf]

nature photonics

Supplementary information

https://doi.org/10.1038/s41566-025-01670-9

Angular dispersion suppression in deeply subwavelength phonon polariton bound states in the continuum metasurfaces

In the format provided by the authors and unedited

Supplementary Information

Contents

1 qBIC in Si and Au negative metasurfaces **2**

2 Transmission FTIR spectra on 100 vs. 200 nm SiC **4**

3 Modulation depth and TO phonon coupling with increasing unit cell number **5**

4 Modeling of bare and coupled SiC metasurface with temporal coupled mode theory **6**

4.1 TCMT framework 6

4.2 TCMT fits to extract qBIC quality factors 7

4.3 TCMT fits for the analysis of vibrational coupling 8

5 Arrow plots of the qBIC resonance and z component phase jumps **11**

6 Simulated transmission under tilted TM and TE excitation **12**

7 Angle dependence for positive SiC and negative Au metasurfaces **13**

8 Numerical study of angle robustness in inverse Drude metasurfaces **14**

9 Simulation of metasurfaces with unit cell size below the fabrication limit **17**

10 PEG thickness estimation **18**

11 Calculation of the dielectric function of PEG **19**

12 Comparison PEG vibrational strength with other molecular vibration for strong coupling **20**

1 qBIC in Si and Au negative metasurfaces

In Fig. 1 of the main text, we compare the SiC metasurface with analogous structures made of elliptical holes in Si and Au films. We fix the dimensions of the unit cells in the metasurfaces to obtain a qBIC resonance at the same frequency as the one for the SiC metasurface at approximately $\lambda = 11.2\,\mu m$. For this, the Au metasurface has a thickness of 100 nm with $S = 3.125$ ($P_x = 9375\,nm$ and $P_y = 6875\,nm$), while for the Si metasurface the film thickness is 1000 nm with $S = 5.36$ ($P_x = 11\,256\,nm$ and $P_y = 8630\,nm$). Simulated spectra for varying ellipses tilt β are shown in Fig. 1S for both materials, with polarization along the short unit cell axis following Babinet's principle. In both cases, a mode arising when breaking the unit cell symmetry arises slightly above 800 cm^{-1} which we individuate as the qBIC mode and is indicated by the blue arrows.

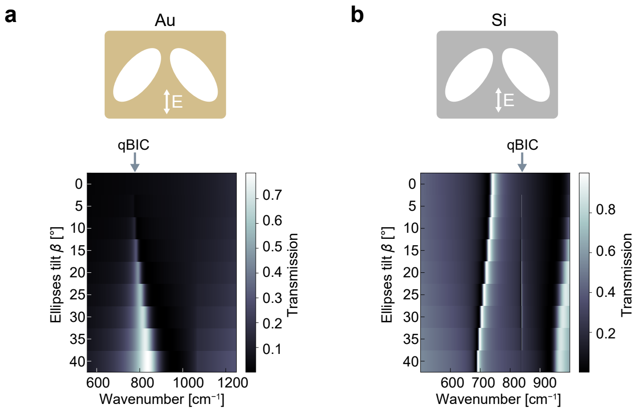

Fig. 1S Simulated spectra for "negative" Au **a** and Si **b** qBIC metasurfaces as a function of the ellipses tilting angle. qBIC modes can be seen arising in both cases at around $\lambda = 11.2\,\mu m$ as indicated by the blue arrows.

For the dispersion plots in Fig. 1 of the main text, we start from the simulations of Fig. 1S with the ellipses at 40° and we tilt the illumination direction. The qBIC can be obtained from both TM polarization (Fig. 2Sa and c for Au and Si respectively) and TE polarization (Fig. 2Sb and d for Au and Si respectively) as long as the unit cell is oriented so that the in-plane projection of the electric field is along the short unit cell axis. In the TM dispersion for Au, the qBIC mode spectrally overlaps with the grating mode that can be seen redshifting in Fig. 2Sa. The two modes couple, causing the jump in the dispersion seen in Fig. 1e of the main text. For the Si metasurface, many peaks appear with tilted illumination, making the tracking of the qBIC mode more complicated. The emergence of many side-peaks is also a drawback in the use of such structure for dielectric metasurfaces, making the interpretation of spectra increasingly complex.

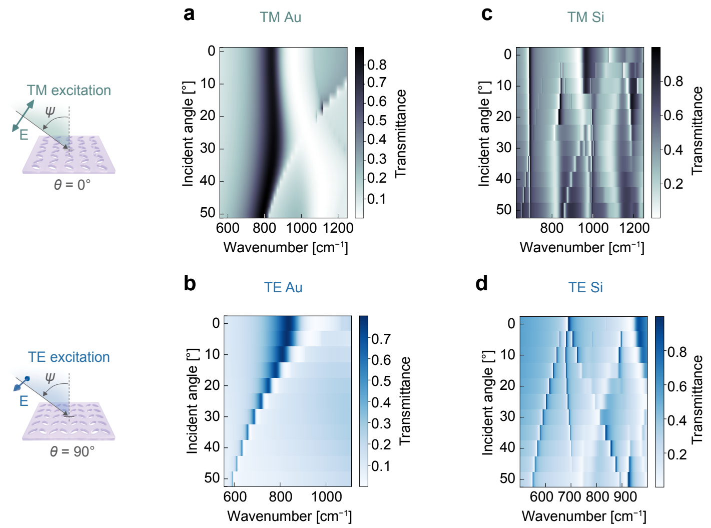

Fig. 2S Simulated spectra for $\beta = 40°$ ellipses tilt at varying incidence angles for TM **a**, **c** and TE **b**, **d** polarizations for the Au and Si "negative" metasurfaces, respectively.

2 Transmission FTIR spectra on 100 vs. 200 nm SiC

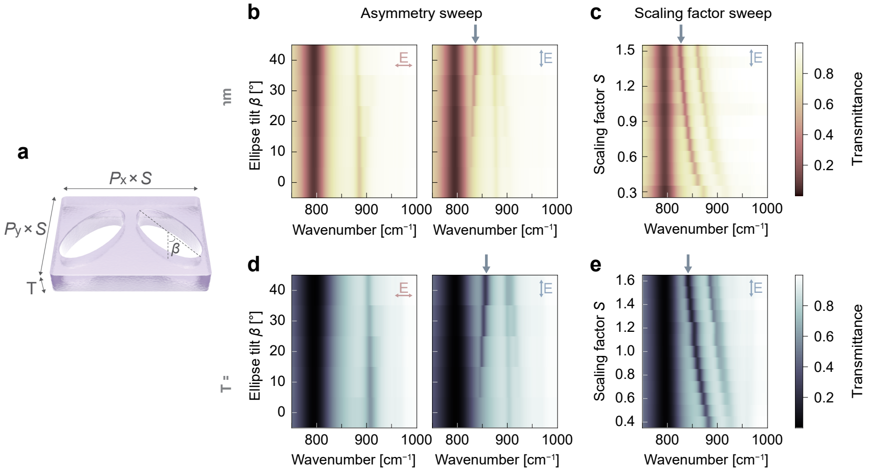

Fig. 3S **a** Schematics of unit cell geometry notations; measured transmittance for asymmetry factor sweep by varying ellipse tilting angle β on **b** 100 nm and d) 200 nm SiC under TE and TM polarization, and scaling factor S sweep on **c** 100 nm and **e** 200 nm SiC under TM polarization. The arrows indicate qBIC modes.

As shown in Fig. 3Sb and d, by increasing the SiC thickness from 100 to 200 nm, the qBIC resonances of the metasurfaces can be blue-shifted. However, the effect of resonance shift from varying the SiC thickness is far less significant than simply changing the scaling factors (Fig. 3Sc and e).

3 Modulation depth and TO phonon coupling with increasing unit cell number

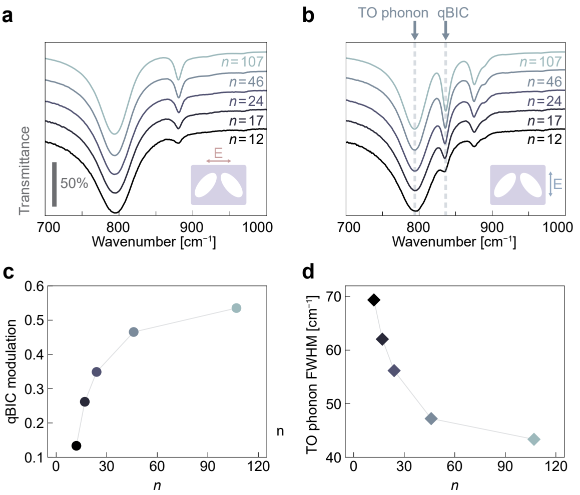

Fig. 4S Experimental transmission spectra of varying numbers of unit cells, n × n, under **a** TE and **b** TM polarization. **c** qBIC resonance modulation depths under TM polarization, and **d** full width half maxima (FWHM) of TO phonon under TM polarization.

The vanishing of qBIC peaks when changing the illumination polarization verifies the typical characteristic behaviour of qBIC metasurfaces. Analysis of modulation depth from experimental transmission spectra for $S = 1$ at $\beta = 40°$, with unit cell number variations, $n \times n$, reveals an increase in modulation with higher unit cell numbers. Additionally, the narrowing of the TO phonon bandwidth suggests coupling between qBIC and TO phonon modes.

4 Modeling of bare and coupled SiC metasurface with temporal coupled mode theory

4.1 TCMT framework

To accurately describe the interplay between the multiple material-intrinsic and photonic resonances of the SiC metasurface, we employ a framework based on temporal coupled-mode theory (TCMT) [1, 2]. The system can be described as a multi-resonant cavity (modelled by a scattering matrix M), which is coupled to two external ports that allow for transmitted and reflected waves. The scattered fields $\mathbf{s}_-$ read

$$\mathbf{s}_- = \mathrm{M}\mathbf{s}_+ \tag{1}$$

with

$$\mathrm{M} = \mathrm{C} + \mathrm{K}\left(i\left(\omega\mathbb{1} - \Omega\right) + \Gamma\right)^{-1}\mathrm{K}^{\mathrm{T}}. \tag{2}$$

Assuming the metasurface is only excited from one port, the transmission coefficient t is given by the matrix element s_{21} of the 2×2 scattering matrix. Here, C denotes non-resonant port coupling

$$\mathrm{C} = e^{i\phi}\begin{pmatrix} r_0 & it_0 \\ it_0 & r_0 \end{pmatrix}, \tag{3}$$

where r_0 and t_0 are reflection and transmission coefficients of the spectral baseline with $r_0^2 + t_0^2 = 1$ and ϕ is a global phase. The cavity modes are described by Ω and Γ, where Ω includes the resonance frequencies, intrinsic damping rates and near-field coupling terms and Γ the radiative damping rates. The coupling of the modes to the far field (ports) is given by K.

Our description of the bare SiC BIC metasurface encompasses four distinct resonances, where the two major ones are the BIC and TO phonon followed by two minor resonances describing SP resonances. When combined with the PEG molecular layer, we split the PEG absorption into two parts, where one part only exhibits intrinsic losses, which couple in the near field with the BIC, mediated by the coupling strength g and one part that shows both intrinsic and radiative losses, which describes the uncoupled surplus molecules (Fig. 5S).

In general, the diagonal elements of Ω are given by $\omega_{jj} = \omega_{0,j} + i\gamma_{\mathrm{int},j}$, where ω_0 is the resonance frequency of the mode and γ_{int} the intrinsic damping rate. Without the presence of a molecular absorption layer the off-diagonal elements are zero. The radiative damping rates γ_{rad} in Γ are given by $\gamma_{jk} = \sqrt{\gamma_{\mathrm{rad},j}\gamma_{\mathrm{rad},k}}$. The mode-port coupling K is described by a $2 \times n$ matrix, where n is the number of modes in the system with $\kappa_{1,j} = \kappa_{2,j} = \sqrt{\gamma_{\mathrm{rad},j}}$.

In the case of vibrational coupling, the first 3×3 block matrices can be written explicitly as

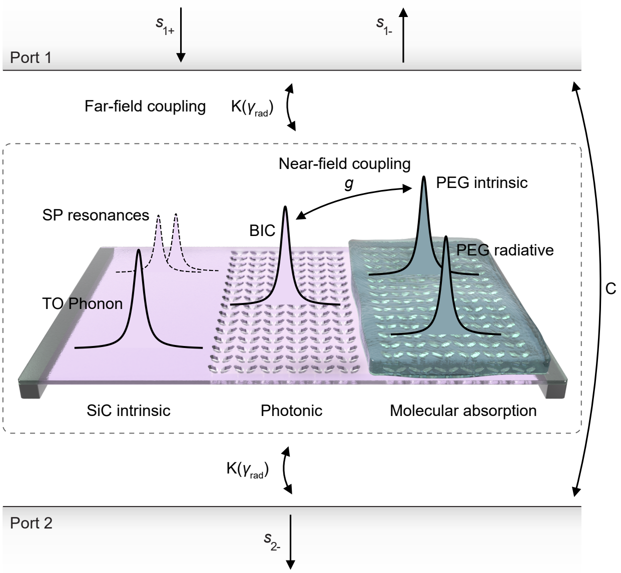

Fig. 5S Schematic representation of the TCMT model describing the SiC BIC metasurface. The system is excited from port 1 (s_{1+}) and the transmitted signal is collected at port 2 (s_{2-}). All resonances in the system (TO phonon, SP resonances, BIC and PEG absorption) exhibit radiative losses, which couple in the far field, contributing to the overall shape of the transmittance spectrum. The coupling strength to the ports is described by K and the near-field coupling of the BIC to the non-radiative part of the PEG absorption band is mediated by the coupling strength g. Non-resonant port coupling is governed by C.

$$\Omega_{\text{cp}} = \begin{pmatrix} \omega_{0,\text{BIC}} + i\gamma_{\text{int,BIC}} & g & 0 \\ g & \omega_{0,\text{PEG}} + i\gamma_{\text{int,PEG}} & 0 \\ 0 & 0 & \omega_{0,\text{PEG}} + i\gamma_{\text{int,PEG}} \end{pmatrix} \tag{4}$$

$$\Gamma_{\text{cp}} = \begin{pmatrix} \gamma_{\text{rad,BIC}} & 0 & \sqrt{\gamma_{\text{rad,BIC}} \cdot \gamma_{\text{rad,PEG}}} \\ 0 & 0 & 0 \\ \sqrt{\gamma_{\text{rad,BIC}} \cdot \gamma_{\text{rad,PEG}}} & 0 & \gamma_{\text{rad,PEG}} \end{pmatrix} \tag{5}$$

$$\text{K}_{\text{cp}} = \begin{pmatrix} \sqrt{\gamma_{\text{rad,BIC}}} & 0 & \sqrt{\gamma_{\text{rad,PEG}}} \\ \sqrt{\gamma_{\text{rad,BIC}}} & 0 & \sqrt{\gamma_{\text{rad,PEG}}} \end{pmatrix}. \tag{6}$$

4.2 TCMT fits to extract qBIC quality factors

By fitting $\mathsf{s\_{-}21''^2} = \mathsf{t}^2 = T$ of equation (2) to transmittance spectra, we can separate the intrinsic and radiative damping rates, and thus the Q factors, of the qBIC resonances. As seen in Fig. 6Sa, the BIC resonance wavenumber does not shift significantly with varying asymmetry. This leads to the valid approximation that the intrinsic damping rate is the same for all asymmetries. By fitting multiple experimental transmittance spectra with a shared intrinsic

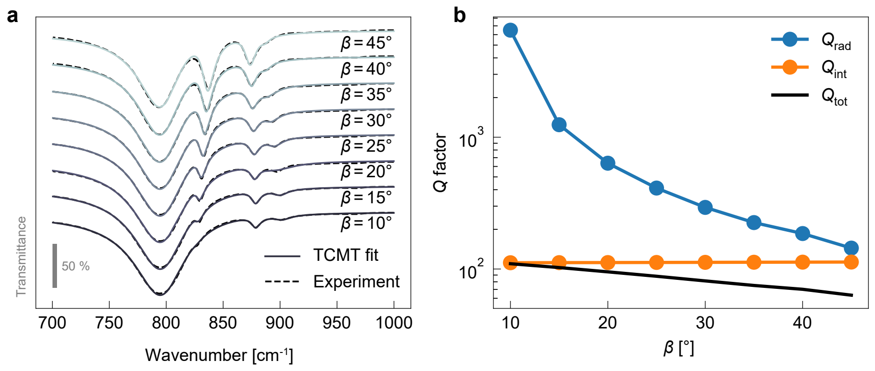

Fig. 6S TCMT fits of experimental asymmetry sweep. **a** Experimental spectra (dashed) and fits (solid) of SiC BIC metasurfaces for different ellipse tilting angles β with shared intrinsic BIC damping rate $\gamma_{\mathrm{int,BIC}}$. **b** Extracted radiative, intrinsic, and total Q factors from panel **a**.

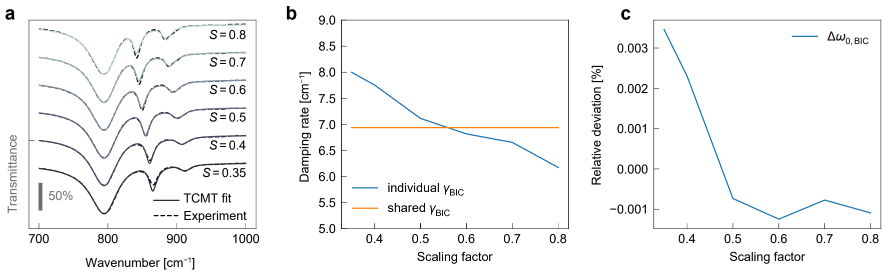

Fig. 7S TCMT fits of multiple spectra with shared and individual BIC damping rates. **a** Experimental transmittance spectra of bare SiC BIC metasurface for multiple scaling factors (dashed) and TCMT fits with over all spectra shared total BIC damping rate γ_{BIC}. **b** Extracted BIC damping rates for fits, where each spectrum is fit with an individual γ_{BIC} and over all spectra shared γ_{BIC}. **c** Relative deviation of resonance wavenumber for fits with shared and individual γ_{BIC} showing values lower than 0.004 %.

BIC damping rate $\gamma_{\mathrm{int,BIC}}$, we can retrieve the BIC-typical inverse square behavior of the radiative quality factor (Fig. 6Sb, Fig. 2d).

4.3 TCMT fits for the analysis of vibrational coupling

In the same manner, we can utilize the complete TCMT model including PEG (Equations (4,5,6)) to analyze the near-field coupling. In order to extract the coupling strength, i.e., the Rabi splitting, and the coupling regime, the knowledge about the i) BIC dispersion, ii) BIC linewidth and iii) PEG absorption linewidth is required. While the latter is easy to obtain experimentally, the first two points are hidden, as the addition of the molecular absorption layer simultaneously alters and masks the BIC properties. The analysis via TCMT provides a way to extract the coupling parameters reliably, using the assumption that the linewidth of the BIC is constant over the sampled scaling factor

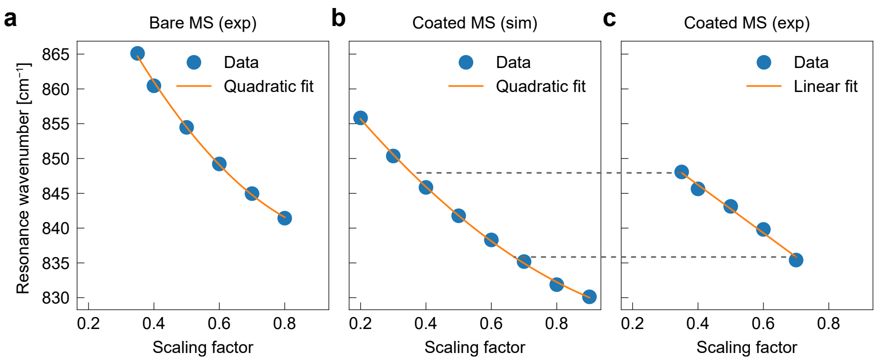

Fig. 8S Comparison of BIC dispersion for bare and coated metasurfaces. **a** Resonance wavenumbers extracted from TCMT fits of experimental transmittance spectra of bare SiC BIC metasurfaces for different scaling factors. The shift of resonance position can be empirically modelled with a quadratic relationship. **b** Resonance wavenumbers from fits of simulated spectra of PEG-coated metasurfaces with a film thickness of 30 nm. The polymer induces a spectral redshift, the overall quadratic behavior remains the same. **c** Resonance wavenumbers of experimental spectra of coated metasurfaces with a film thickness of 30 nm. Due to slight fluctuations of the thickness on metasurfaces of different scaling factors, the wavenumbers fluctuate and can be better approximated with a linear relationship, which is a good approximation in the chosen sampling range of scaling factors in experiment.

range. As seen in Fig. 7S, at the example of measured transmittance spectra of a bare 100 nm thick SiC BIC metasurface, shifting the qBIC resonance towards the TO phonon changes the total BIC damping rate γ_{BIC}. By sharing γ_{BIC} over multiple spectra, we essentially average the damping rate to a value that corresponds to the center spectrum in the sampled range. This approximation is in line with conventional coupling fit approaches, such as direct fits of polariton branches, where a constant cavity linewidth is usually assumed. While the linewidth deviates from a chosen center point, the extracted resonance wavenumbers remain identical (Fig. 7Sc), demonstrating the capability of the TCMT model to accurately extract the BIC dispersion.

This capability also extends to PEG-coated metasurfaces, where the bare BIC signal is not directly readable (Fig. 8S). Because of thickness fluctuations of the PEG layer in experiment, we approximate the BIC dispersion of the coupled spectra with a linear function (see main text).

With above assumptions we can now analyze the experimental transmittance spectra of the with 30 nm thick PEG-coated SiC BIC metasurfaces for different scaling factors (Fig. 9S). The fitted spectra are centered around the scaling factor $S = 0.5$ that shows best overlap between the BIC and PEG absorption band. Because we share the fit parameter γ_{BIC} among all spectra, we can extract the BIC linewidth at the scaling factor of optimal coupling. We furthermore share the resonance wavenumber and linewidth of the TO phonon and fix the PEG parameters from measurements to reduce the number of free parameters. We can then evaluate the scaling factor of optimal coupling by intersecting the BIC dispersion with the resonance wavenumber of the PEG

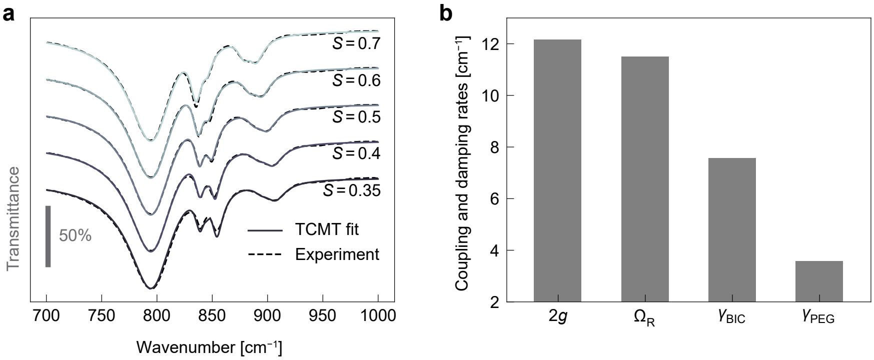

Fig. 9S Vibrational coupling analysis of 30 nm PEG-coated SiC BIC metasurfaces. **a** Experimental transmittance spectra and fits for different scaling factors. **b** Extracted coupling and damping rates, clearly showing that the peak separation exceeds the damping rates of BIC and PEG.

absorption band, yielding $S = 0.435$. We subsequently evaluate all parameters required to analyze the coupling regime at this scaling factor, as can be found in the main text. By solving the eigenvalue problem of the first 2×2 block matrix defined in Equation (4), we can find an expression for the polariton dispersion

$$\omega_{\pm} = \frac{\omega_{\mathrm{BIC}} + \omega_{\mathrm{PEG}}}{2} + \frac{i(\gamma_{\mathrm{BIC}} + \gamma_{\mathrm{PEG}})}{2} \pm \sqrt{g^2 - \frac{1}{4}\left(\gamma_{\mathrm{BIC}} - \gamma_{\mathrm{PEG}} + i(\omega_{\mathrm{BIC}} - \omega_{\mathrm{PEG}})\right)^2} \tag{7}$$

from which we can extract the Rabi splitting

$$\Omega_{\mathrm{R}} = (\omega_{+} - \omega_{-})_{\omega_{\mathrm{BIC}} = \omega_{\mathrm{PEG}}} = 2\sqrt{g^2 - \frac{1}{4}\left(\gamma_{\mathrm{BIC}} - \gamma_{\mathrm{PEG}}\right)^2}. \tag{8}$$

Comparing the individual damping rates of BIC and PEG with the Rabi splitting Ω_{R} and its hypothetical maximum for $\gamma_{\mathrm{BIC}} = \gamma_{\mathrm{PEG}}$, $2g$, we clearly see that the dip splitting exceeds the damping rates, as required for strong coupling.

As can be seen from Fig. 4b, overlaying the polariton dispersion calculated from parameters extracted from the TCMT fit shows excellent agreement with both simulation and experiment, underlining the validity of our fitting approach.

5 Arrow plots of the qBIC resonance and z component phase jumps

To understand why the z component of the electric field at the qBIC resonance has phase jumps in the opposite direction of the exciting polarization (see Fig. 2 of the main text), it is useful to plot the simulated 3D field. In Fig. 10S, we report simulated data for a metasurface with $\beta = 30°$ at the qBIC resonance. The exciting polarization is along the $y-$axis. In Fig. 10Sa, the phase of the out of plane field E_z is shown with characteristic phase jumps in the direction orthogonal to the polarization of the exciting beam. In Fig. 10Sb, an arrow plot of the field at the top surface shows how the in-plane fields in the elliptical holes resemble two slightly tilted opposite horizontal dipoles. The side view in Fig. 10Sc reveals how the fields go in and out from the opposite sides of the elliptical holes, which results in a change of sign of E_z along the short ellipses axis and horizontal jumps in the field phase. The same trend can also be observed in the substrate surrounding the elliptical holes. The same E_z behaviour is also confirmed by the perspective view of Fig. 10Sc.

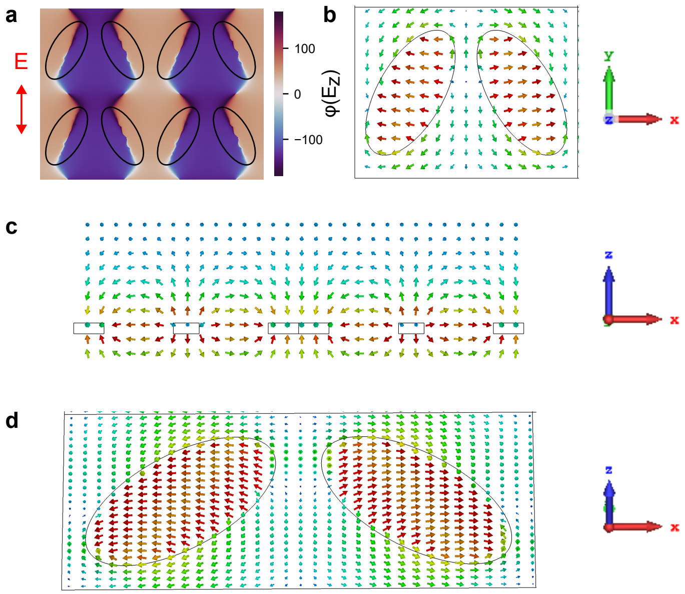

Fig. 10S Snapshots of the simulated field of a SiC metasurface with $\beta = 30°$ and $S = 1$ at the qBIC frequency. **a** E_z phase map. **b** Top view at the upper SiC surface. **c** Side view in the middle of the ellipses. **d** Perspective view rotated around the $x-$axis

6 Simulated transmission under tilted TM and TE excitation

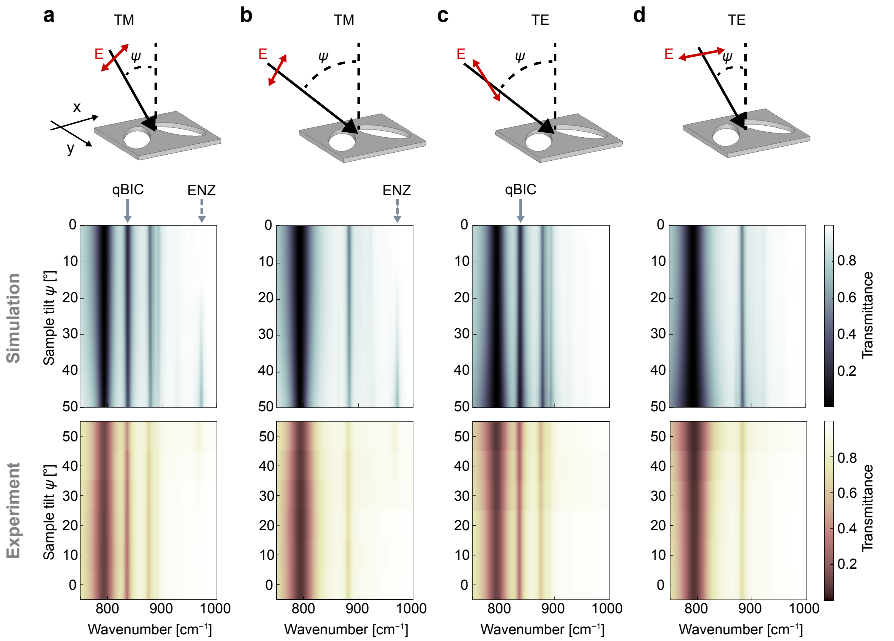

Fig. 11S Simulated and experimental transmission spectra for various incident angles ψ for **a**, **b** TM and **c**, **d** TE polarization. For each polarization, we plot both cases where the in-plane projection of the electric field is along the **a**, **c** $y-$ and **b**, **d** $x-$axis.

Simulated and experimental transmission spectra at various incident angles, ψ, for a metasurface with $\beta = 40°$ and $S = 1$. Highlighted by the solid arrows are the dips corresponding to the qBIC resonances (only when the polarization is along the $y-$axis) and by the dashed arrows are the Brewster mode (only for TM polarization) at the epsilon-near-zero (ENZ) point.

7 Angle dependence for positive SiC and negative Au metasurfaces

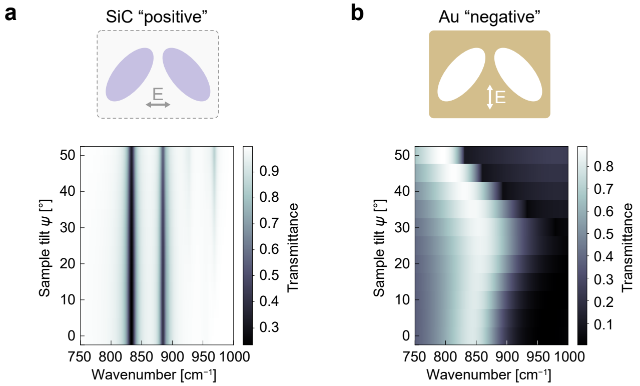

Fig. 12S Simulated angle-dependent transmission for **a** a "positive" SiC metasurface ($S = 1, \beta = 40°$) and **b** a "negative" Au metasurface ($S = 3.1, \beta = 40°$). In both cases, the film thickness is 100 nm.

We show here with simulations that the angle-independent behaviour of our SiC metasurface can be attributed to the deeply subwavelength unit cell size and not by its specific geometrical arrangement. For comparison, an identical "positive" metasurface made of SiC antennas also features resonances that do not shift when tilting the sample with respect to the incident beam is shown in Fig. 12Sa. On the other hand, a "negative" metasurface made of holes milled in an Au film shows a broad resonance that is significantly dispersive with ψ. Note that in Fig. 12Sb, the unit cell is much larger than for the SiC case ($S = 3.1$ for the Au and $S = 1$ for the SiC metasurface) in order to have a qBIC resonance at the SiC RS band frequency. As the Au film is completely reflective to mid-IR radiation, the resonance appears as a peak in the transmission and not as a dip.

8 Numerical study of angle robustness in inverse Drude metasurfaces

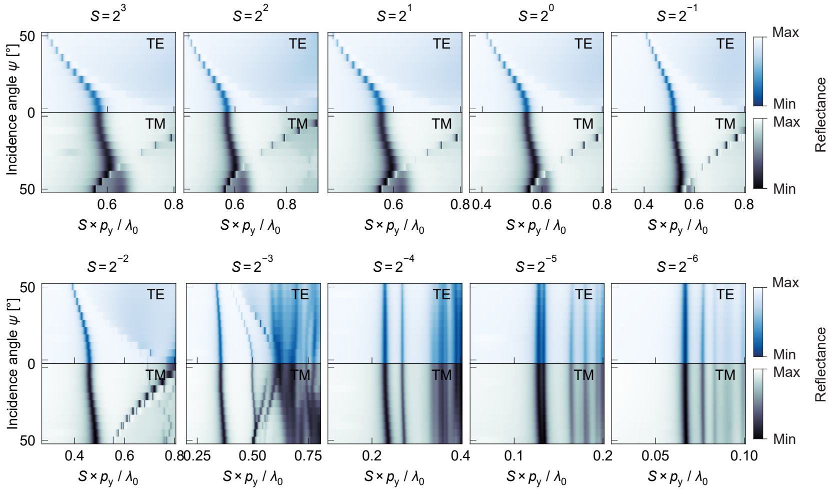

Fig. 13S TE and TM BIC branches of Drude metasurfaces for different scaling factors plotted against the degree of unit cell subwavelengthness.

To further corroborate our findings of angle independence linked to the deeply subwavelength nature of our metasurface, we simulate inverse BIC metasurfaces with structural parameters identical to our SiC design using an artificial Drude material with a permittivity given by

$$\varepsilon(\omega) = 1 - \frac{\omega_{\mathrm{p}}^2}{\omega(\omega + i\gamma)}, \tag{9}$$

where we chose a plasma frequency of $\omega_{\mathrm{p}} = 2000\,\mathrm{THz}$ and a damping rate $\gamma = 140\,\mathrm{THz}$. With these parameters, we reach the SiC RS band frequency for scaling factors of around 4. We systematically reduce the scaling factor by a factor of 2 while keeping the resonator height constant and simulate the reflectance spectra for different incidence angles for both TE and TM excitations (Fig. 13S). Using a Drude material has the advantage of simpler mode structures given by the absence of phonon peaks while retaining the property of a strongly dispersive and negative real part of the permittivity, although at different spectral regimes. Given that Maxwell's equations are scale invariant, we introduce the degree of subwavelengthness described by the ratio of unit cell periodicity in excitation direction and free space wavelength $S \times P_{\mathrm{y}}/\lambda_0$. We quantified the degree of dispersion by taking the average of the absolute derivative of the qBIC branches over an incidence angle range between 0° and 50° using spline interpolation.

For most scaling factors, the quasi-BIC resonances at normal incidence appear at a subwavelengthness of between 0.5 and 0.6, where both TE and TM qBIC branches show a strongly dispersive behavior. Reducing the scaling factor leads to a sudden drop in subwavelengthness which is accompanied by a reduction of dispersion, where TE and TM bands become eventually angle-robust for subwavelengthnesses below 0.2 (Fig. 14S).

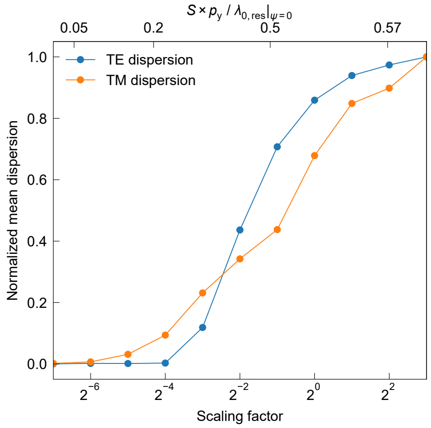

Fig. 14S Normalized mean dispersions of TE and TM branches for different scaling factors and degrees of subwavelengthness respectively. Both exhibit strong dispersive behavior for a wide scaling factor range and a sudden drop of dispersion below a threshold of sufficiently small scaling factors.

The reduction of subwavelengthness can be explained by a saturation of resonance position for scaling factors below 2^{-4} corresponding to the spectral range where the Drude material approaches the polaritonic regime (see main text). In the metallic regime, the qBICs exhibit the typical linear scaling behavior (Fig. 15Sa). Importantly, the BIC-inherent control over the resonance linewidth is retained for even extremely low subwavlengthnesses one order of magnitude smaller than for metallic BICs (Fig. 15Sb).

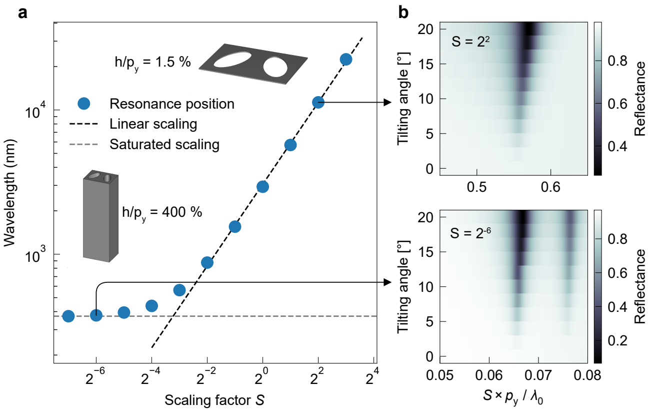

Fig. 15S Scaling behavior of Drude BIC metasurfaces. **a** Absolute resonance position with respect to the unit cell scaling factors. In the metallic regime, the BIC resonance positions follow a typical linear scaling law. For very small scaling factors corresponding to the plasmonic regime of the material, resonance positions saturate. **b** Simulated reflectance spectra for different tilting angles in metallic and plasmonic regimes confirming the qBIC enabled control over resonance linewidth even for extremely subwavelength metasurfaces.

9 Simulation of metasurfaces with unit cell size below the fabrication limit

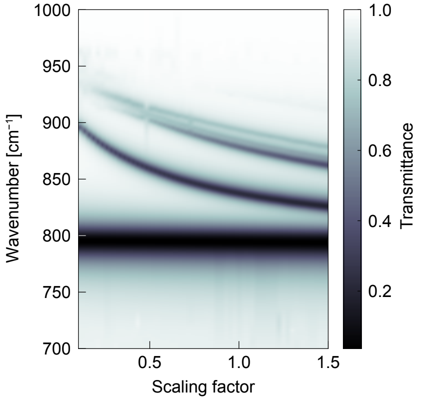

Fig. 16S Simulated transmission spectra for a SiC metasurface with $\beta = 40°$ and varying scaling factor from $S = 0.05$ to $S = 1.5$

For large unit cells we observe a saturation of the frequency position towards the TO phonon. A similar effect is expected at high energies towards the LO phonon, but is harder to observe because of the extremely small unit cell size required. In Fig. 16S, we show simulated transmission spectra for a SiC metasurface where we still observe a continuous blue-shift of the qBIC resonance with decreasing S even when the scaling factor down to $S = 0.05$, which corresponds a minimum lateral unit cell size of 80 nm.

10 PEG thickness estimation

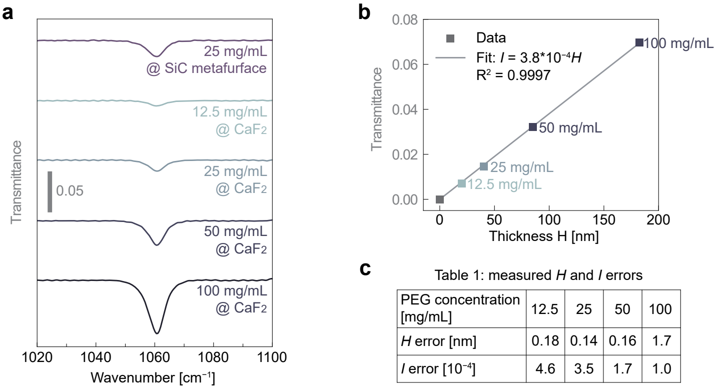

Table 1: measured *H* and *I* errors

PEG concentration [mg/mL]	12.5	25	50	100
H error [nm]	0.18	0.14	0.16	1.7
I error [10^{-4}]	4.6	3.5	1.7	1.0

Fig. 17S **a** Measured transmittance of PEG on SiC metasurface and CaF_2 substrates, **b** fitted correlation in between PEG transmittance and thickness at band $1060\,\mathrm{cm}^{-1}$, and **c** standard error in ellipsometry and FTIR measurements.

The thickness-transmittance relationship was acquired via ellipsometry and transmission FTIR measurements. When the thickness of polymer itself is smaller than the penetration depth of the incident wavelength, the absorbance of the film is solely dependent on the number of the molecules that are being excited by the incident wavelength. From this, we can assume a simple linear relationship in between the transmission of the thin film and its thickness: $I = kH$[3], where H is the height of the thin film, and I is the transmittance. To estimate k, PEG solution with various concentration was spin-coated on single-polished Si and CaF_2 substrates under exactly the same spin-coating conditions followed by the same surface cleaning and treatment procedures. The thicknesses of the polymer was primarily confirmed to be similar on Si and CaF_2 with stylus profilometer. For more accurate assessment, the thickness of PEG was measured with ellipsometry on Si substrates, and the thicknesses were correlated with the measured transmission intensities at peak around $1060\,\mathrm{cm}^{-1}$ that is acquired from transmission FTIR measurement. The characteristic band at $1060\,\mathrm{cm}^{-1}$ corresponds to C-O-C symmetrical stretching. This band is chosen because it is far outside the range of any modes of the metasurfaces. From the correlation curve, it is possible to inter- and extrapolate the actual thickness of the PEG on SiC metasurfaces.

11 Calculation of the dielectric function of PEG

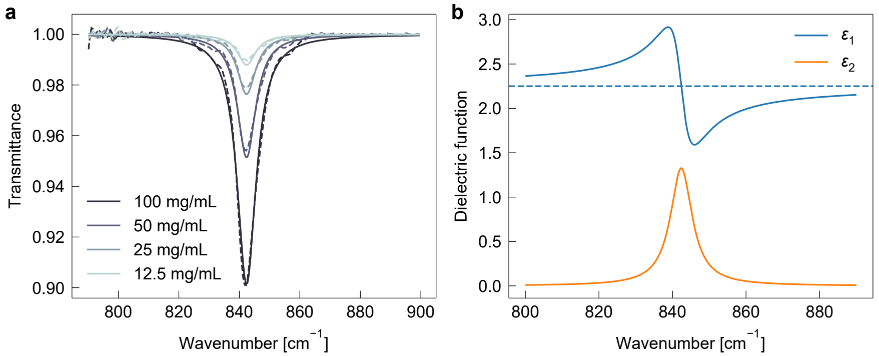

Fig. 18S Extraction of dielectric function of PEG from transmission measurements. **a** Measured transmission spectra of PEG films of different concentrations on CaF_2 substrates (dashed) and fits using the Tinkham formula (solid). **b** Dielectric function of PEG, calculated with parameters extracted from the fits in panel **a**.

We approximate the dielectric function of PEG around 842 cm^{-1} by fitting the Tinkham formula[4] to transmission measurements in the respective spectral range. The transmittance of a thin film on a substrate with respect to its dielectric function $\epsilon(\omega)$ is given by

$$T = \mid 1 - \frac{idZ_0\varepsilon_0\omega\,(\varepsilon(\omega) - 1)}{1 + n} \mid^{-2}, \tag{10}$$

where d is the thickness of the film, Z_0 and ε_0 are the vacuum impedance and permittivity, ω is the frequency and n the refractive index of the substrate. We describe the dielectric function of PEG using a Lorentzian oscillator model[5]

$$\varepsilon(\omega) = \varepsilon_\infty + \frac{A}{\omega_0^2 - \omega^2 - 2i\gamma\omega}, \tag{11}$$

where ω_0 denotes the resonance frequency, γ the half width at half maximum and A the oscillator strength of the molecular vibration band.

We extract these properties by fitting equation (10) to multiple transmittance spectra of PEG of different thicknesses (see section 10) on CaF_2 substrates (Fig. 18S), where we approximate the refractive index of CaF_2 to be 1.25 in the considered spectral range[6]. This yields following parameters describing the absorptive properties of the molecule $\omega_0 = 842.4\,\text{cm}^{-1}$, $\gamma = 3.59\,\text{cm}^{-1}$ and $A_{\text{PEG}} = 8026\,\text{cm}^{-2}$. The non-resonant real part of the permittivity $\varepsilon_{\text{PEG},\infty}$ is set to 2.25, which provides excellent agreement of numerical simulations with experiments.

12 Comparison PEG vibrational strength with other molecular vibration for strong coupling

The appearance of vibrational strong coupling for a coupled resonator-molecule system depends both on the properties of the cavity and of the target molecule. The PEG peak we use in our work is relatively weak compared to other vibrational modes used to reach the strong coupling regime. To make a comparison, consider a molecular vibration described by a Lorentz model as defined in Equation (11).

To reach the strong coupling regime, the following inequality must hold for the target vibrational mode:

$$\mathrm{FOM} = \frac{A}{\varepsilon_\infty \gamma} > 1 \tag{12}$$

which corresponds to the existence of bulk polariton modes in a semi-infinite slab. We can then define the left hand of the above equation as a figure of merit indicating how easy it is to reach the strong coupling regime with a certain molecule. Another metric of comparison is the bulk coupling strength at zero detuning defined as $g_B = 0.5\sqrt{A/\varepsilon_\infty}$. A final metric can be defined through the condition to achieve ultra-strong coupling $\eta = g/\omega_0 \geq 0.1$.

	ε_∞	A (cm^{-2})	ω_0 (cm^{-1})	γ (cm^{-1})	FOM (cm^{-1})	g_B (cm^{-1})	η
PMMA[7]	1.99	$6.38 \cdot 10^5$	1732	9.87	$3.2 \cdot 10^4$	282	0.16
CBP[8]	2.2	$2.5 \cdot 10^4$	1450	4.1	$2.73 \cdot 10^3$	53	0.037
SiO_2[9]	2.1	$3.3 \cdot 10^5$	1046	0.75	$2.03 \cdot 10^5$	198	0.19
PEG	2.25	$8.0 \cdot 10^3$	842	3.59	993	30	0.035

Table 2 Molecular resonator strengths vs. coupling strengths on previously reported data

References

[1] Haus, H.A.: Waves and fields in optoelectronics. Prentice-Hall (1984)

[2] Suh, W., Wang, Z., Fan, S.: Temporal coupled-mode theory and the presence of non-orthogonal modes in lossless multimode cavities. IEEE J. Quantum Electron. **40**(10), 1511–1518 (2004)

[3] Yang, P., Meng, X., Zhang, Z., Jing, B., Yuan, J., Yang, W.: Thickness measurement of nanoscale polymer layer on polymer substrates by attenuated total reflection infrared spectroscopy. Analytical Chemistry **77**(4), 1068–1074 (2005)

[4] Li, P., Dolado, I., Alfaro-Mozaz, F.J., Esteban, R., Atxabal, A., Casanova, F., Hueso, L.E., Alonso-González, P., Aizpurua, J., Nikitin, A.Y., *et al.*:

Boron nitride nanoresonators for phonon-enhanced molecular vibrational spectroscopy at the strong coupling limit. Light: Science & Applications **7**(4), 17172–17172 (2018)

[5] Canales, A., Karmstrand, T., Baranov, D.G., Antosiewicz, T.J., Shegai, T.O.: Polaritonic linewidth asymmetry in the strong and ultrastrong coupling regime. Nanophotonics **12**(21), 4073–4086 (2023)

[6] Li, H.H.: Refractive index of alkaline earth halides and its wavelength and temperature derivatives. Journal of Physical and Chemical Reference Data **9**(1), 161–290 (1980)

[7] Arul, R., Menghrajani, K., Rider, M.S., Chikkaraddy, R., Barnes, W.L., Baumberg, J.J.: Raman probing the local ultrastrong coupling of vibrational plasmon polaritons on metallic gratings. Phys. Rev. Lett. **131**, 126902 (2023)

[8] Bylinkin, A., Schnell, M., Autore, M., Calavalle, F., Li, P., Taboada-Gutierrez, J., Liu, S., Edgar, J.H., Casanova, F., Hueso, L.E., *et al.*: Real-space observation of vibrational strong coupling between propagating phonon polaritons and organic molecules. Nature Photonics **15**(3), 197–202 (2021)

[9] Yoo, D., de León-Pérez, F., Pelton, M., Lee, I.-H., Mohr, D.A., Raschke, M.B., Caldwell, J.D., Martín-Moreno, L., Oh, S.-H.: Ultrastrong plasmon–phonon coupling via epsilon-near-zero nanocavities. Nature Photonics **15**(2), 125–130 (2021)
